# Prevalence of measles in vaccinated and non-vaccinated children

**DOI:** 10.17179/excli2015-170

**Published:** 2015-04-01

**Authors:** Muhammad Asif Zahoor, Muhammad Hidayat Rasool, Muhammad Waseem, Bilal Aslam, Muhammad Kashif Zahoor, Muhammad Saqalein, Zeeshan Nawaz, Rabia Sahar

**Affiliations:** 1 Department of Microbiology, Govt. College University, Faisalabad-Pakistan; 2 Department of Zoology, Wild Life and Fisheries, Govt. College University, Faisalabad-Pakistan

## ⁯

Dear Editor,

Measles is a highly infectious and contagious disease of the respiratory system caused by *Morbilivirus* which belongs to family *Paramyxoviridae *(Hashiguchi et al., 2011[8]). The disease is a common cause of childhood morbidity and mortality across the globe, particularly in developing countries and has been characterized by high fever, cough, conjunctivitis, coryza, malaise and maculopapular rash along with erythematous patches throughout the body (Ellison, 1931[4]; Yanagi et al., 2006[21]; Fazlalipour et al., 2008[5]). Measles infection has been controlled by introduction of live attenuated measles vaccine in United States and Europe (Gindler et al., 2004[7]). However, measles is still affecting the developing countries due to insufficient coverage and improper handling of vaccines (Poland and Jocobson, 1994[15]; Muscat et al., 2009[14]). Historically, immunization against vaccine preventable diseases (VPDs) in children has been started by WHO in 1974, and it was initiated in Pakistan during 1978 (Ali, 2000[1]; Bugvi et al., 2014[2]). In general the vaccine coverage against VPDs in Pakistan ranged between 56 to 88 % which significantly varied among various Provinces (Sheikh et al., 2011[19]). 

Recently, increased number of measles outbreaks with high morbidity and mortality has been observed in various regions of Pakistan during recent years (Khan and Khan, 2012[10]; Khan and Qazi, 2014[11]). These particular episodes of higher incidence of measles were started in Thatta, Mirpurkhas and Jacobabad Districts of Sindh Province by the end of 2012, which later on spread towards Punjab Province (Khan and Khan, 2012[10]; Khan and Qazi, 2014[11]). The spread of measles was reported to be higher in both rural as well as developed cities of Pakistan along with high incidence of mortality and morbidity (Khan and Qazi, 2014[11]). The important factors considered for these outbreaks were associated with vaccination failure due to several reasons i.e. low vaccination coverage, malnutrition and vitamin-A deficiencies, poor vaccination facilities in remote and rural areas, mis-handling of vaccines and lack of immunization awareness among parents due to lower levels of education in various areas of country (Cohen et al., 2009[3]; Khan and Khan, 2012[10]; Khan and Qazi, 2014[11]). To combat the situation extensive supplementary immunization activities have been initiated which targets the children less than 10 years of age particularly in Punjab, Pakistan under Expanded Program on Immunization (EPI). 

Therefore, in the current investigation, impact of supplementary vaccination has been estimated in children from Faisalabad and Jhang using enzyme linked Immunosorbent assay (ELISA) as outlined in supplemental material (). A total of 871/1053 (82.71 %) children from Faisalabad and 647/813 (79.58 %) children from Jhang were found vaccinated either with single or dual dose of measles vaccination (Table 1(Tab. 1)). Out of these 264 blood samples from vaccinated and 100 samples from non-vaccinated children were collected randomly and analysed for the presence of anti-measles IgG antibodies. Only 73.48 % of vaccinated children developed humoral immune response as detected through ELISA. This indicated that the protection against measles was not optimum according to WHO standards (Rabenau et al., 2007[16]; Fazlalipour et al., 2008[5]; Lauridan and van Damme, 2007[12]). Sero conversion following vaccination against measles in developing countries has been reported to be lower (75 %) due to certain factors as described previously (Fowotade et al., 2013[6]; Shah et al., 2012[18]). The highest values (95 %) for measles vaccination have been reported in European countries (Tischer and Gerike, 2000[20]).

Samples from non-vaccinated children showed high prevalence (63 %) which was an indication of previous measles infection in these particular children. These findings were suggestive and may be correlated with confirmatory sero-diagnosis of recent outbreaks in these areas. The non-vaccination status against measles was considered as one of the major risk factor in children (Khan and Qazi, 2014[11]). 

The prevalence of anti-measles IgG antibodies from samples collected from Faisalabad was found 79.54 % whereas in Jhang 67.42 % of samples were observed as positive. There was no significant difference of sero-prevalence between Faisalabad and Jhang. The possible explanation for non-significant prevalence could be that these two areas are closely related geographically, traditionally and are closely situated to each other. These factors may be considered for a similar trend towards vaccination coverage and sero-conversion against measles as previously described by Hussain et al. (2008[9]). 

The prevalence of anti-measles IgG antibodies from samples collected from male children was higher as compared to female children with non-significant difference. However, few of the available literature has reported that risk of measles and level of anti-measles IgG antibodies was observed higher in females as compared to male children (Rahim et al., 2011[17]; Fowotade et al., 2013[6]; Bugvi et al., 2014[2]). Therefore, from the current findings, it may be concluded that gender has no relation with associated risk factors of measles. 

Based on various age groups, prevalence of anti-measles IgG antibodies were observed as 75.35 % in children from 1-6 years of age, whereas 67.56 % in children from 6-10 years of age were positive with no significant difference. These findings were not in accordance with some of the previous studies which showed the highest incidence of measles during the age of 6 months to 3 years (Matsumura et al., 2005[13]; Rahim et al., 2011[17]). The possible explanation for the observed trend in the current investigation might be due to recent outbreaks during 2012-13 as previously reported (Khan and Qazi, 2014[11]) or very extensive SIAs during 2013 against measles which possibly resulted in development of increased humoral immune response in children up to the age of 10 years.

It was concluded from the overall results of present study that vaccination coverage against measles was below the standards of WHO and Health Department, Govt. of Punjab, Pakistan. Further, vaccine efficacy and development of humoral immune response in children was not optimum according to WHO guidelines and higher risk of measles incidence was found in non-vaccinated children. Based on these findings, it is recommended that routine immunization against all vaccine preventable diseases in general and against measles in particular should be carried out as per guidelines of WHO to completely control and eradicate measles from Pakistan.

## Conflict of interest

The authors declare that they have no conflict of interest.

## Supplementary Material

Supplementary material

## Figures and Tables

**Table 1:**
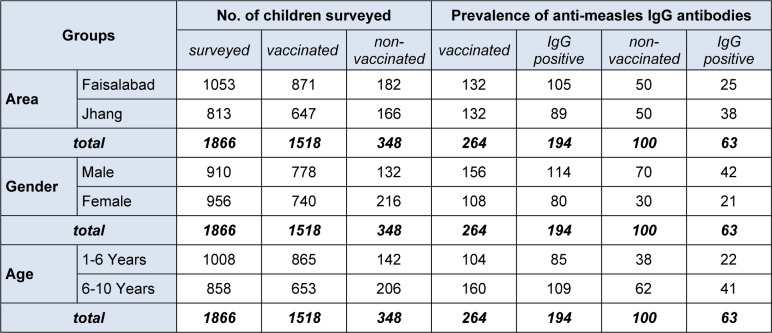
Vaccination coverage and prevalence of anti-measles IgG antibodies in vaccinated and non-vaccinated children of Faisalabad and Jhang, Pakistan
