# Supplementary material for: Prevalence of measles in vaccinated and non-vaccinated children
Source: EXCLI J. 2015 Apr 1;14:504–7. doi: 10.17179/excli2015-170 (PMC4667567; doi:10.17179/excli2015-170)
Supplement: Supplementary material [file EXCLI-14-504-s-001.pdf]

EXCLI Journal 2015;14:504-507 – ISSN 1611-2156
Received: January 30, 2015, accepted: March 27, 2015, published: April 01, 2015

Supplementary material to:

Letter to the editor:

PREVALENCE OF MEASLES IN VACCINATED AND NON-VACCINATED CHILDREN

Muhammad Asif Zahoor[1]*, Muhammad Hidayat Rasool[1], Muhammad Waseem[1], Bilal Aslam[1], Muhammad Kashif Zahoor[2], Muhammad Saqalein[1], Zeeshan Nawaz[1,] Rabia Sahar[1]

[1] Department of Microbiology, Govt. College University, Faisalabad-Pakistan
[2] Department of Zoology, Wild Life and Fisheries, Govt. College University, Faisalabad-Pakistan

* Corresponding author: Muhammad Asif Zahoor Department of Microbiology, Govt. College University, Faisalabad-Pakistan. Tel: 92-41-9201205;
E-mail address: drasifzahoor@gcuf.edu.pk

http://dx.doi.org/10.17179/excli2015-170

This is an Open Access article distributed under the terms of the Creative Commons Attribution License (http://creativecommons.org/licenses/by/4.0/).

MATERIALS AND METHODS

A brief questionnaire was designed to collect all necessary information regarding area, gender, age and history of vaccination or measles infection.

Collection of samples and serum separation

From Faisalabad, a total of 1053 children under 10 years of age were surveyed from September, 2013 to May, 2014 about vaccination and disease history of measles. Out of these, 182 blood samples (132 from vaccinated and 50 from non-vaccinated individuals) were collected randomly. Similarly during the same period, from Jhang, 813 children under 10 years of age were surveyed during the same period about vaccination and disease history of measles. Out of these, 182 blood samples (132 from vaccinated and 50 from non-vaccinated individuals) were collected randomly. The blood was collected from each individual in 2.5 or 5ml disposable syringes. Each blood sample was transferred immediately to separate serum clot activator tube (Lab Vac Gel and Clot Activators), kept on ice and shifted to Department of Microbiology, Govt. College University, Faisalabad. Serum was separated from each blood sample by centrifugation of serum collection tubes at 1000-2000 Xg for 3-5 minutes and stored in 1.5ml Eppendorf tubes at -20 $^{\circ}$C.

Enzyme linked immunosorbent assay

Enzyme Linked Immunosorbent Assay (ELISA) was performed (Etchart et al., 2007; Fazlalipour et al., 2008) under optimized conditions for the determination of anti-measles IgG antibodies (humoral immune response) using commercially available ELISA kit (NovaLisa ™, Measles Virus IgG - ELISA, Nova Tec Immunodiagnostica GmbH) according to manufacturer's instructions. Briefly, individual serum samples were diluted with serum diluent and 100µl of each diluted serum sample was added in each well of ELISA plate along with blank,

negative control, positive control and cut-off control. Following incubation of ELISA plate for one hour at 37 °C, plate was washed with 300 µl of washing buffer. In the next step, 100 µl measles anti-IgG conjugate was added into all wells except for the blank well, and kept for 30 minutes at room temperature. Finally, 100 µl tetramethylbenzidine substrate (TMB) was added into all wells, and kept at room temperature for 15 minutes in dark. Reaction was stopped by using 100 µl of stop solution into all wells. Absorbance of the ELISA plate was determined at 450 nm within 30 minutes after addition of the stop solution (Biotek®, ELX 808, USA).

Statistical analysis

The data obtained by measuring the absorbance values of each well of ELISA plate was arranged in Microsoft Excel spread sheet for each group i.e. vaccination status, area, gender and age. Absorbance values in each group were compared with cut-off value. The anti-measles IgG data from each groups was compared statistically using chi-square test using Minitab® 16.1 (Dagan et al., 1995; Ozbek et al., 1999; Rabenau et al 2007).

REFERENCES

Dagan R, Slater PE, Duvdevani P, Golubev N, Mendelson E. Decay of maternally derived measles antibody in a highly vaccinated population in southern Israel. Pediatr Infect Dis J. 1995;14:965-9.

Etchart N, Hennino A, Friede M, Dahel K, Dupouy M, Henry CG, et al. Safety and efficacy of transcutaneous vaccination using a patch with the live-attenuated measles vaccine in humans. Vaccine. 2007;25:6891-9.

Fazlalipour M, Monavari SH, Shamsi SM, Ataei A. Evaluation of immune status to measles in vaccinated population in Tehran, by enzyme-linked immunosorbent assay and the hemagglutination inhibition techniques (1386-1387). Iran J Virol. 2008;2:27-30.

Ozbek S, Vural M, Tastan Y, Kahraman I, Perk Y, Ilter O. Passive immunity of premature infants against measles during early Infancy. Acta Paediatr. 1999;88: 1254-7.

Rabenau HF, Marianov B, Allwinn R. Comparison of the neutralizing and ELISA antibody titres to measles virus in human sera and in gamma globulin preparations. Med Microbiol Immunol. 2007;196:151–5.
